# Production of latex agglutination reagents for pneumococcal serotyping

**DOI:** 10.1186/1756-0500-6-49

**Published:** 2013-02-05

**Authors:** Belinda D Ortika, Maha Habib, Eileen M Dunne, Barbara D Porter, Catherine Satzke

**Affiliations:** 1 Pneumococcal Research, Murdoch Childrens Research Institute, Royal Children’s Hospital, Parkville, Victoria, Australia; 2 Infectious Diseases and Microbiology, Murdoch Childrens Research Institute, Royal Children’s Hospital, Parkville, Victoria, Australia; 3 Department of Microbiology and Immunology, The University of Melbourne, Parkville, Victoria, Australia

**Keywords:** Latex agglutination, Serotyping, *Streptococcus pneumoniae*

## Abstract

**Background:**

The current ‘gold standard’ for serotyping pneumococci is the Quellung test. This technique is laborious and requires a certain level of training to correctly perform. Commercial pneumococcal latex agglutination serotyping reagents are available, but these are expensive. In-house production of latex agglutination reagents can be a cost-effective alternative to using commercially available reagents. This paper describes a method for the production and quality control (QC) of latex reagents, including problem solving recommendations, for pneumococcal serotyping.

**Results:**

Here we describe a method for the production of latex agglutination reagents based on the passive adsorption of antibodies to latex particles. Sixty-five latex agglutination reagents were made using the PneuCarriage Project (PCP) method, of which 35 passed QC. The other 30 reagents failed QC due to auto-agglutination (n=2), no reactivity with target serotypes (n=8) or cross-reactivity with non-target serotypes (n=20). Dilution of antisera resulted in a further 27 reagents passing QC. The remaining three reagents passed QC when prepared without centrifugation and wash steps. Protein estimates indicated that latex reagents that failed QC when prepared using the PCP method passed when made with antiserum containing ≤ 500 μg/ml of protein. Sixty-one nasopharyngeal isolates were serotyped with our in-house latex agglutination reagents, with the results showing complete concordance with the Quellung reaction.

**Conclusions:**

The method described here to produce latex agglutination reagents allows simple and efficient serotyping of pneumococci and may be applicable to latex agglutination reagents for typing or identification of other microorganisms. We recommend diluting antisera or removing centrifugation and wash steps for any latex reagents that fail QC. Our latex reagents are cost-effective, technically undemanding to prepare and remain stable for long periods of time, making them ideal for use in low-income countries.

## Background

To date, over 90 capsular serotypes of *Streptococcus pneumoniae* (the pneumococcus) have been described [1,2]. Current vaccines target pneumococcal capsular polysaccharide and protect against serotypes responsible for the majority of invasive disease [3,4]. However, widespread vaccination may lead to the replacement of vaccine serotypes by non-vaccine serotypes [5,6]. Serotyping is an important tool for evaluating the long-term effectiveness of pneumococcal vaccines and providing broader epidemiological information [7]. The ‘gold standard’ pneumococcal serotyping method is the Quellung reaction [8,9]. This microscope-based method is labor intensive, requiring skill and experience to perform and as a result it is usually conducted in reference laboratories [10,11].

Several alternate phenotypic and genotypic pneumococcal serotyping methods have been developed [10,12]. In low-income settings, the most common method is latex agglutination [13-15]. Latex agglutination is simple and rapid to perform, and the results are relatively easy to interpret. This technology is also used to identify other microorganisms, including *Cryptococcus* spp. [16], *Staphylococcus aureus*[17] and rotavirus [18].

Latex agglutination reagents are created by passively adsorbing antibodies to polystyrene latex particles. Commercial pneumococcal serotyping latex reagents have been developed by the Statens Serum Institut (SSI) [14] however these are expensive and do not differentiate all known pneumococcal serotypes. Alternatively, in-house reagents are easy to prepare and use small amounts of antisera, making them ideal for serotyping in low-income settings. This paper details a method for the production and quality control of latex agglutination reagents for pneumococcal serotyping, including problem solving recommendations.

## Methods

### Bacterial isolates

Colonizing and invasive pneumococcal isolates were obtained from a number of geographical regions, including low-income countries. Isolates were identified as *S. pneumoniae* by optochin susceptibility, bile solubility and Phadebact Pneumococcal test kit (Bactus AB, Huddinge, Sweden). Serotypes were determined by the Quellung reaction [9]. Isolates were stored in skim milk-tryptone-glucose-glycerol (STGG) media [19] at −80°C. Immediately prior to testing, isolates were cultured onto horse blood agar plates and incubated for 18–24 h in 5% CO_2_ at 37°C.

### Preparation of latex agglutination reagents

Commercially available pneumococcal factor antisera (Statens Serum Institut, Copenhagen, Denmark) were used to prepare latex reagents using the following method.

### PneuCarriage project (PCP) method

The method of Lafong and Crothers [20] was used with several modifications, namely: dilution of antisera and polystyrene latex beads, incorporation of centrifugation and wash steps, and use of a higher concentration of bovine serum albumin (BSA). Briefly, the antiserum was diluted 1/40 with glycine buffered saline (GBS; 1 M NaCl, 0.1 M glycine, pH 8.2). Polystyrene latex beads (0.8 μm, Sigma-Aldrich, Buchs, Switzerland) were diluted 1/10 in physiological saline. Equal volumes of diluted antiserum and latex suspension were mixed and incubated at 37°C on a rotating wheel for 2 h at approximately 0.01 × *g*. Two rounds of centrifugation (15 min at 1100 × *g*), each followed by washing in GBS, were performed. Equal volumes of GBS and GBS containing 0.2% BSA (w/v) (pH 8.2) were added to a final volume of 4 ml. Sodium azide was added to a final concentration of 1% (w/v) as a preservative. Latex reagents were stored at 4°C.

### Quality control (QC) of latex agglutination reagents

Latex reagents were brought to room temperature. Firstly, a visual check was conducted to ensure the suspension was smooth and white. Each reagent was pipetted onto a glass slide and rotated for one min to ensure auto-agglutination was not present. Then, half a 1 μl loop-full of fresh overnight culture was quickly and thoroughly mixed with 15 μl of latex reagent. The slide was rotated for one min with results observed using an indirect light source over a dark background.

The reagent was tested against a panel of isolates representing target and non-target serotypes based on the SSI key to pneumococcal antisera [21]. At least one isolate of each target and non-target serotype was included in testing. Where there was only one target or non-target serotype in the SSI key, two different isolates were tested. Colonizing isolates were included where possible.

**Figure 1:**
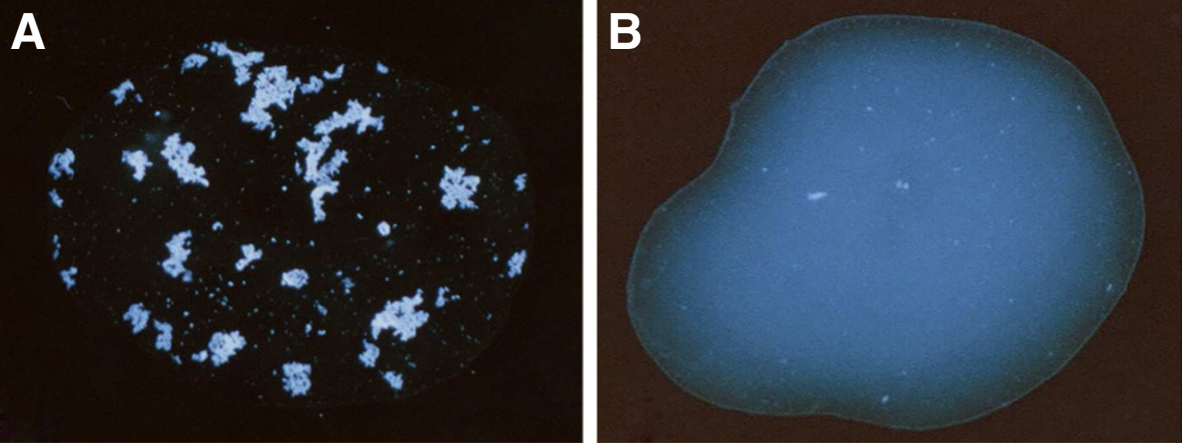
**Latex agglutination test.** A positive reaction (**A**) has visible agglutination and clearing, whilst a negative reaction (**B**) is smooth and white.

Reagents passed QC if the target serotype reactions displayed agglutination and clearing of the suspension, while non-target serotype reactions remained smooth and white (Figure 1). Reactions that were stringy, or agglutinated weakly around the edge of the drop, were considered negative. QC of latex reagents was performed 12 months after production and then every 6 months.

### Protein assay

BCA protein assays (Pierce Biotechnology, Rockford, USA) were performed to determine the protein concentration of the antisera, and were conducted as per the manufacturer’s instructions.

## Results and discussion

Overall, a total of 65 latex agglutination reagents were made using the PCP method, of which 35 passed QC. The other 30 reagents failed QC due to auto-agglutination (n=2), no reactivity with target serotypes (n=8) or cross-reactivity with non-target serotypes (n=20).

Several experiments were conducted to address the causes of QC failure for some latex reagents. These included prior washing of the latex particles to remove interfering surfactant groups [22] and changing the pH and concentrations of buffer solutions to reduce non-specific agglutination [14,23]. However, these approaches did not alter the performance of the latex reagents (data not shown).

BCA protein assays were performed to compare the protein concentrations of antisera in reagents that consistently passed and failed QC (23b and 6b respectively). The concentration of protein in the antiserum was higher for the reagent that failed QC (929.7 μg/ml) compared to the one that passed (207.5 μg/ml). This observation led us to perform doubling dilutions of antisera ranging from 1/80 to 1/1280 to prepare latex reagents that previously failed QC (n=30). Diluting the antisera resulted in 27 of these latex reagents passing QC.

**Figure 2:**
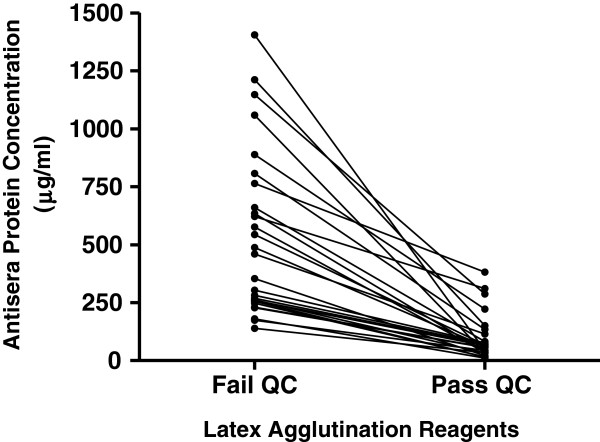
**Protein concentration of antisera in latex agglutination reagents.** Each paired data point represents the protein concentration (μg/ml) of antisera used to prepare latex reagents with the PCP method that failed or passed quality control (QC) procedures.

To determine if there was an optimal protein concentration, BCA protein assays were performed on a number of antisera. Figure 2 shows all 27 latex reagents that failed QC when prepared using the PCP method subsequently passed when made with antiserum containing ≤ 500 μg/ml of protein. Our results indicate that when antisera concentrations below this value are used to prepare latex reagents, they are more likely to result in a QC pass. Interestingly, of the 27 latex reagents that failed QC, 19 passed when made with antiserum containing ≤ 76 μg/ml of protein, suggesting that further dilution of antiserum may be beneficial. Our results also indicate that protein concentrations vary between different antisera, and between different lot numbers of the same antiserum.

Three reagents prepared using the PCP method with antisera dilutions failed QC (7e, 19b and 28b). These reactions displayed weak agglutination that was not accompanied by clearing of the suspension with target serotypes. Based on the publication by Severin [24] we removed the centrifugation and wash steps thereby reducing the possibility of disrupting the bound antibody from the latex particle and increasing the strength of agglutination and clearing. To prepare these three reagents we followed Severin’s method with some modifications, namely, a different antisera dilution, a 1/10 dilution of polystyrene latex beads and GBS containing 0.2% BSA. This approach resulted in the three reagents passing QC.

To assess the accuracy of the latex factor agglutination reagents post-optimization, serotyping was performed on 61 randomly selected nasopharyngeal isolates from developing countries, specifically The Gambia (n=1), Fiji (n=12), Papua New Guinea (n=12), Bangladesh (n=17) and Kenya (n=19). The isolates were also tested using the Quellung reaction. Of the 61 isolates, 21 serotypes from 13 serogroups were identified. These represented serotypes 9V, 15A, 18A, 19B, 22F, 35A and 41A (n=1 of each serotype), 6D, 9N, 10A and 17F (n=2 of each serotype), 6B, 6C, 11A, 16F and 19A (n=3 of each serotype), 35B (n=4), 6A (n=5), 23F (n=6), 15B/C (n=7) and 19F (n=9). Latex agglutination and Quellung serotyping results were in concordance with each other for all 61 isolates.

Given the importance of these reagents, it would be prudent to QC latex reagents regularly to ensure they remain stable and reactive. In our laboratory, latex reagents stored at 2 to 8°C, continued to pass QC for a minimum of two years (data not shown). Given this, and the current lack of long-term data, we suggest reagents be retested after an initial 12 month period, then every 6 months.

The cost of producing our latex factor reagents is $13.77 USD per bottle (4 ml) and $0.06 USD per test (15 μl), excluding labor and consumable costs. In our laboratory it takes approximately 4 h to produce and QC twelve latex reagents, and the QC consumable costs are approximately $2.10 USD per bottle. Most reagents (35/65) passed QC with a single dilution, for the remainder the mean number of dilutions required was 3.1 (95% CI: 2.6, 3.5).

In order to produce in-house latex reagents a collection of antisera is required. Laboratories that are conducting Quellung will already have this resource available. Given that the cost of commercial latex reagents range from approximately $1.26-1.94 USD per test, and that the cost of each Quellung test (1 μl) in our laboratory is $0.21 USD, in-house latex reagents may be a cost effective option for laboratories routinely performing serotyping.

## Conclusions

Overall, a total of 65 latex agglutination reagents were produced and validated for the serotyping of pneumococci. We recommend the PCP method starting with a 1/40 dilution of antisera for producing latex agglutination reagents. For reagents that fail QC, two problem solving approaches should be taken. The first approach is to further dilute the antisera. If this is unsuccessful, the second approach of removing centrifugation and wash steps should be applied. Protein estimates may be useful when attempting to make a latex reagent with a new lot number of antisera, indicating what dilution to use in order to obtain a reagent that passes QC.

The method described here to produce latex agglutination reagents allows simple and efficient serotyping of pneumococci. Although this paper describes the production of reagents using individual factor antisera, we have successfully created reagents with type and pooled antisera also (data not shown). Our method may be applicable to latex reagents for typing or identification of other microorganisms. Our latex reagents are cost-effective, technically undemanding to prepare and remain stable for long periods of time, making them ideal for use in low-income countries.

## Competing interests

The authors declare that they have no competing interests.

## Authors’ contributions

BO and MH contributed to the overall study design, performed the experimental work, data analysis and wrote the manuscript, ED contributed to the overall study design and manuscript preparation, BP provided technical oversight, and contributed to the overall study design and manuscript preparation, CS led the overall study design and manuscript preparation. All authors read and approved the final manuscript.
